# Hospitalized Versus Outpatient Benign Acute Childhood Myositis: A 10-Year Single-Center Experience

**DOI:** 10.3390/medicina62030583

**Published:** 2026-03-20

**Authors:** Yasemin Özkale, Murat Özkale, Şeyda Beşen, Tuba Karsantıözü, Nihal Aktaş, Gökçe Yegül Gülnar, Burak Poyraz

**Affiliations:** 1 Department of Pediatrics, Faculty of Medicine, Dr Turgut Noyan Teaching and Medical Research Center, Baskent University, 01140 Adana, Turkey; 2 Department of Pediatric Intensive Care, Faculty of Medicine, Dr Turgut Noyan Teaching and Medical Research Center, Baskent University, 01140 Adana, Turkey; 3 Department of Pediatrics, Division of Child Neurology, Faculty of Medicine, Dr Turgut Noyan Teaching and Medical Research Center, Baskent University, 01140 Adana, Turkey

**Keywords:** benign acute childhood myositis, creatine kinase, hospitalization, influenza

## Abstract

*Background and Objectives*: The objectives of this study were to compare the clinical, laboratory, and etiological characteristics, as well as outcomes, of hospitalized and outpatient children with benign acute childhood myositis (BACM) and to identify factors associated with hospitalization. *Materials and Methods*: This retrospective single-center study included children diagnosed with BACM over a 10-year period. Demographic data, clinical features, laboratory parameters, etiological agents, treatments, hospitalization status, and recurrence were analyzed. Hospitalized and outpatient patients were compared to determine factors associated with hospital admission. *Results*: A total of 93 patients were included. Hospitalized patients had significantly higher creatine kinase (CK) levels and a higher frequency of inability to walk compared with outpatients. No significant differences were observed between the groups regarding age, sex, gastrointestinal symptoms, serum creatinine levels, or inflammatory markers. Influenza A (INFA)-associated BACM was characterized by lower CK levels and shorter fever duration, whereas viral panel negative (VPN) cases had longer symptom duration and were more frequently hospitalized. Notably, sandfly fever virus was identified in two hospitalized patients, representing an uncommon but clinically relevant etiological agent in our cohort. Rhabdomyolysis occurred in three patients, all of whom were hospitalized and recovered without sequelae. The recurrence rate was 11.8%, with no significant association between recurrence and demographic or clinical variables. *Conclusions*: Although BACM is typically self-limiting, elevated CK levels and inability to walk may help identify patients who require hospitalization. Etiological differences influenced disease severity, and the detection of sandfly fever-associated BACM highlights the importance of considering regional viral agents in the differential diagnosis.

## 1. Introduction

Benign acute childhood myositis (BACM), also referred to as influenza-associated myositis or acute viral myositis, is a transient inflammatory condition of skeletal muscle characterized by acute bilateral calf pain, muscle tenderness, and difficulty walking. It typically develops within two to four days following a viral infection and predominantly affects school-aged children, with a higher incidence in males [1,2]. BACM may occur sporadically or in epidemic clusters, most commonly during the winter and spring seasons.

Epidemic cases are most frequently associated with influenza viruses, particularly *influenza B (INFB)*, whereas sporadic cases have been linked to a wide range of viral and bacterial pathogens. Reported etiological agents include *parainfluenza virus*, *SARS-CoV-2*, *Epstein–Barr virus* (EBV), *cytomegalo virus (CMV)*, *human herpesvirus 6 (HHV6)*, *respiratory syncytial virus (RSV)*, *Coxsackie viruses* *Mycoplasma pneumoniae*, *Streptococcus pyogenes*, *Legionella* species, and *Salmonella* species [1,2,3]. The etiopathogenesis of BACM remains unclear; proposed mechanisms include direct viral invasion of muscle tissue, cytokine-mediated muscle injury, and immune-mediated inflammatory responses.

Clinically, BACM typically presents with sudden-onset lower extremity pain and gait disturbance, often leading to significant parental anxiety and urgent medical evaluation. Diagnosis is primarily based on characteristic clinical findings supported by laboratory abnormalities, most notably elevated serum creatine kinase (CK) levels. Transient cytopenias and mild elevations in lactate dehydrogenase (LDH), aspartate aminotransferase (AST), and alanine aminotransferase (ALT) levels may also be observed [2,3,4,5]. Because BACM can mimic serious neurological, rheumatological, or malignant conditions, accurate diagnosis is essential to avoid unnecessary investigations and interventions.

Although BACM is generally self-limiting and resolves with supportive care, hospitalization may be required in selected cases due to severe symptoms or concerns regarding potential complications [2,6]. Previous studies have primarily focused on clinical features and etiological agents; however, data comparing hospitalized and outpatient patients and identifying objective criteria for hospitalization remain limited. Furthermore, etiological distributions vary according to geographic region and study design, and data from Turkey are scarce.

Therefore, the primary aim of this study was to evaluate the clinical and laboratory characteristics of children diagnosed with BACM at a tertiary care center in southern Turkey and to identify factors associated with hospitalization by comparing hospitalized and outpatient patients. As a secondary objective, we aimed to provide region-specific data on the viral etiologies of BACM, reflecting local patterns of viral circulation.

## 2. Materials and Methods

### 2.1. Study Design and Patient Selection

This retrospective single-center study was conducted at Başkent University Adana Hospital. The medical records of children and adolescents diagnosed with benign acute childhood myositis (BACM) at the Departments of Pediatric Emergency Medicine, Pediatrics, and Pediatric Neurology between January 2015 and January 2025 were reviewed.

A total of 2246 patients identified using the International Classification of Diseases (ICD) codes M60, M72, M79, and R26 were initially screened. Among these, 102 patients met the preliminary diagnostic criteria for BACM. Nine patients were excluded due to the diagnosis of a neuromuscular disorder during follow-up. Consequently, 93 patients were included in the study.

The diagnostic criteria for BACM were defined as follows: (1) acute onset of bilateral lower extremity pain and/or inability to walk; (2) occurrence after prodromal symptoms such as fever, cough, coryza, sore throat, nausea, vomiting, or weakness; (3) normal neurological examination findings; and (4) elevated serum creatine kinase (CK) levels.

Patients were excluded if their symptoms were attributable to alternative diagnoses, including traumatic muscle injury, excessive physical activity, drug-induced myopathy, pyomyositis, autoimmune myositis, neuromuscular or metabolic disorders, myocarditis, prematurity, post-resuscitation status, epilepsy, dermatomyositis, or other chronic neuromuscular diseases.

### 2.2. Data Collection

Demographic, clinical, and laboratory data at the time of initial presentation were obtained from the hospital’s electronic medical record system. Collected variables included age, sex, presenting symptoms, etiological agent, treatment modality, recovery time, hospitalization status, recurrence, and clinical outcomes.

Laboratory parameters included complete blood count (CBC), C-reactive protein (CRP), serum CK, CK-MB, LDH, AST, ALT, serum creatinine, urinalysis findings, and follow-up CBC and CK values.

Serum analyses were performed using chemoluminescent microparticle immunoassay with a commercial kit (Abbott Laboratories, Abbott Park, IL, USA) on an Abbott Architect i2000 system (Abbott Laboratories). Reference ranges were defined as follows: CK, 30–150 U/L; AST, 5–35 U/L; ALT, 6–46 U/L; LDH, 160–450 U/L; and serum creatinine, 0.3–1.1 mg/dL.

Rhabdomyolysis was defined as the presence of myoglobinuria accompanied by serum creatine kinase (CK) levels ≥ 5 times the upper limit of normal (ULN). Acute kidney injury was defined according to the Kidney Disease: Improving Global Outcomes (KDIGO) criteria.

### 2.3. Virological and Metabolic Evaluation

Available virological data were retrospectively reviewed. These included results of respiratory viral panel testing (*influenza A*, *Influenza B*, *RSV*, and *SARS-CoV-2*) and, when clinically indicated, serum serological testing for *EBV*, *CMV*, *HSV*, and *Mycoplasma pneumoniae*. The viral panel negative (VPN) group was defined as patients who tested negative for respiratory viral pathogens included in the diagnostic panel and in whom no viral or atypical bacterial etiology could be identified through additional serological evaluation. Thus, the VPN group represents cases with undetermined etiology. In patients with recurrent episodes of BACM, metabolic investigations were reviewed to exclude underlying metabolic disorders. These evaluations included tandem mass spectrometry, urine organic acid analysis, and plasma amino acid profiling.

### 2.4. Treatment and Follow-Up

Data regarding patient management were recorded, including follow-up setting (outpatient or hospitalized), treatment modalities (analgesics, intravenous hydration, urine alkalinization, antiviral therapy, and renal replacement therapy), and duration of hospitalization.

Patients were categorized into hospitalized and outpatient groups. Demographic, clinical, laboratory, and etiological characteristics were compared between these groups to identify factors associated with hospitalization. Patients were also categorized according to etiological agents into four groups: VPN, *INFA*, *INFB*, and other etiologies, including *EBV*, *SFV*, *and* viral co-infections (e.g., *INFA + RSV* and *INFA + HSV*).

Follow-up procedures were defined as follows. For hospitalized patients, recovery was defined as resolution of symptoms accompanied by normalization or a downward trend in CK levels documented during inpatient monitoring. For outpatients, recovery time was determined retrospectively based on findings documented at follow-up visits, typically performed approximately one week after the initial presentation, during which symptom resolution was recorded in the medical records.

The study was approved by the Institutional Review Board and Ethics Committee of Başkent University (approval number: KA25/323). Written informed consent was obtained from the parents or legal guardians of all participants.

## 3. Statistical Analysis

All statistical analyses were performed using SPSS software version 27.0 (SPSS Inc., Chicago, IL, USA). The distribution of continuous variables was assessed for normality using the Kolmogorov–Smirnov test.

Normally distributed continuous variables were presented as the mean ± standard deviation, whereas non-normally distributed variables were expressed as the median and interquartile range (minimum–maximum). Categorical variables were summarized as frequencies and percentages.

Comparisons between hospitalized and outpatient patients were performed using Student’s *t*-test for normally distributed continuous variables and the Mann–Whitney U test for non-normally distributed variables. Comparisons among more than two independent groups were conducted using a one-way analysis of variance (ANOVA), followed by post hoc analyses to identify pairwise group differences when appropriate.

Categorical variables were compared using the chi-square test or Fisher’s exact test, as appropriate, based on expected cell counts. Logistic regression analysis was performed to identify factors independently associated with hospitalization and recurrence in patients with BACM. Variables with a *p* value < 0.10 in univariate analyses were entered into the multivariable logistic regression model. Because CK values were highly skewed and typically ranged in the thousands, CK levels were log-transformed and rescaled to represent 1000 U/L increases in the logistic regression analysis to improve the interpretability of the effect size. Results were reported as odds ratios (ORs) with 95% confidence intervals (CIs). A two-tailed *p* value < 0.05 was considered statistically significant.

## 4. Results

### 4.1. Demographic Characteristics

**Figure 1:**
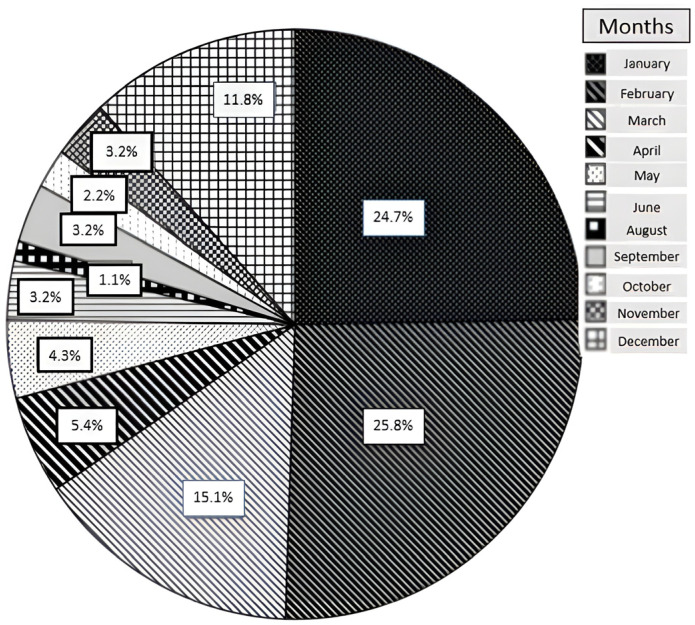
Monthly distribution of benign acute childhood myositis (BACM) cases during the study period.

**Figure 2:**
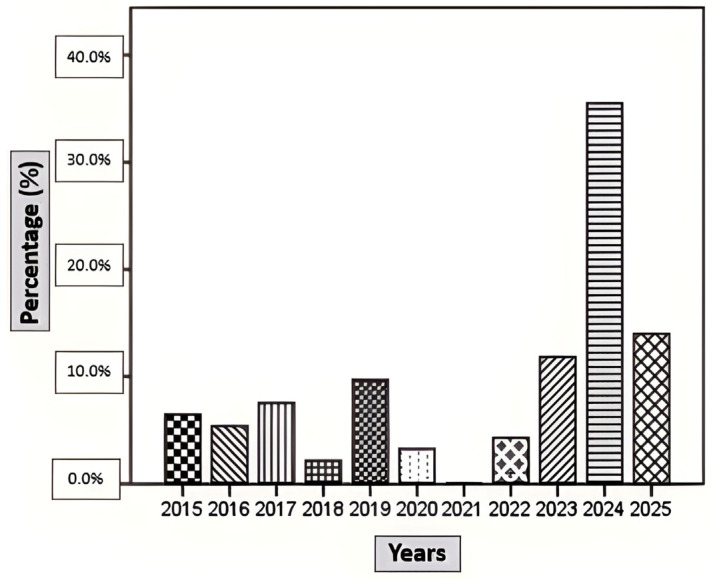
Annual distribution of benign acute childhood myositis (BACM) cases during the study period.

**Figure 3:**
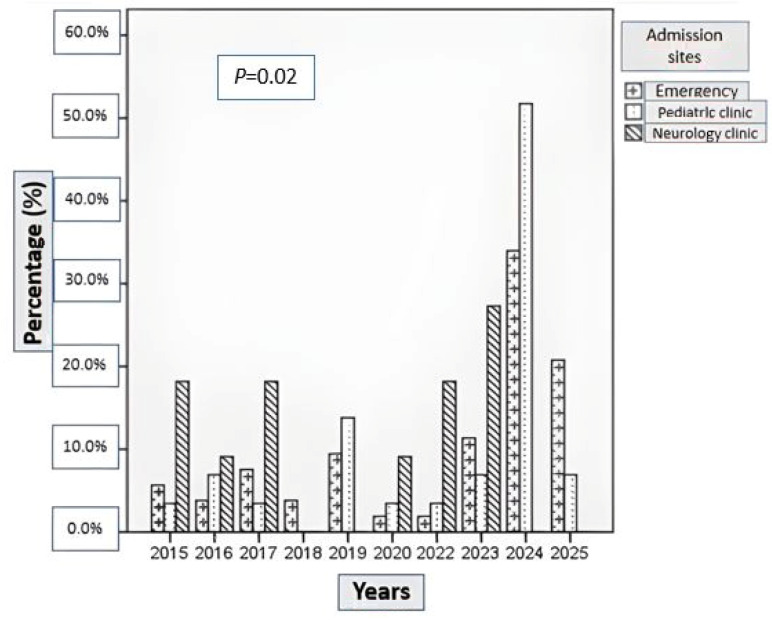
Distribution of admission sites for patients with benign acute childhood myositis (BACM) by year of admission.

The demographic characteristics of patients with BACM are summarized in Table 1. A total of 93 patients were included in the study, of whom 51 (54.8%) were hospitalized and 42 (45.2%) were managed on an outpatient basis. The monthly and annual distributions of cases, as well as admission sites, are shown in Figure 1, Figure 2 and Figure 3. BACM was most frequently diagnosed during the winter months, particularly in January (24.7%), February (25.8%), and March (15.1%). During the 10-year study period, the highest number of admissions occurred in 2024, accounting for 35.4% of all cases.

### 4.2. Comparison of Hospitalized and Outpatient Patients

**Figure 4:**
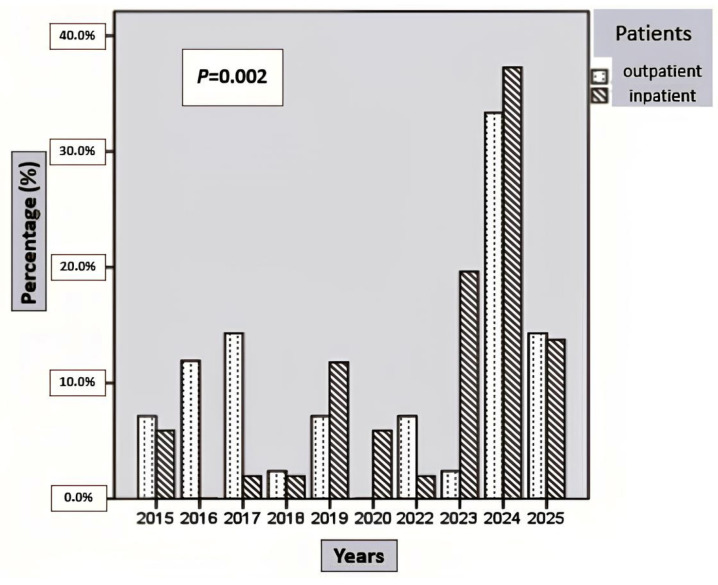
Annual distribution of hospitalized and outpatient patients with benign acute childhood myositis (BACM). The proportion of hospitalized and outpatient patients differed significantly across years (*p* = 0.002).

No significant difference was observed in the monthly distribution of cases between hospitalized and outpatient patients (*p* > 0.05). In both groups, admissions peaked in January (hospitalized, 25.5%; outpatient, 23.8%) and February (hospitalized, 25.5%; outpatient, 26.2%) (Table 2). In the years 2019, 2020, 2023, and 2024, the proportion of hospitalized patients was significantly higher than that of outpatients (Figure 4) (*p* < 0.05).

Comparisons of demographic and clinical characteristics between hospitalized and outpatient patients are presented in Table 2 and Table 3. There were no significant differences between the groups in terms of mean age, sex, duration of fever, cough, upper respiratory tract infection (URTI) symptoms, sore throat, myalgia, gastrointestinal symptoms (including diarrhea, abdominal pain, nausea, and vomiting), headache, conjunctival redness, postnasal drip, or the interval between fever onset and myalgia onset (*p* > 0.05). In contrast, inability to walk at presentation was significantly more frequent among hospitalized patients compared with outpatients (*p* = 0.0001).

### 4.3. Laboratory Findings

Laboratory findings of hospitalized and outpatient patients are summarized in Table 3. No significant differences were observed between the groups with respect to hemoglobin levels; leukocyte, neutrophil, or lymphocyte counts; serum creatinine levels; CRP; or LDH levels (*p* > 0.05). However, mean aspartate AST, ALT, and CK levels were significantly higher in hospitalized patients compared with outpatients (*p* < 0.05). In addition, mean platelet counts were significantly lower in hospitalized patients (*p* < 0.05). Follow-up CK levels remained significantly higher in hospitalized patients compared with outpatients (*p* = 0.01).

### 4.4. Etiological Comparisons

Etiological testing was not performed in 31 of the 93 patients. In these patients, the diagnosis of BACM was established based on clinical findings and elevated CK levels; therefore, they were excluded from the etiological analysis. Among the remaining 62 patients who underwent etiological evaluation, *INFA* was the most frequently detected pathogen in the outpatient group (45.5%), whereas VPN cases were more common among hospitalized patients (50.0%) (*p* < 0.05). Sandfly fever virus was identified in two hospitalized patients who presented during the summer months.

Comparisons among these groups revealed significant differences in CK levels, fever duration, myalgia duration, and the interval between fever onset and myalgia onset (*p* < 0.05). No significant differences were observed with respect to recovery time or duration of inability to walk (Table 4).

To further explore differences between etiological groups, pairwise post hoc comparisons were performed following the overall analysis. Median CK levels were significantly lower in the *INFA* group than in both the VPN (*p* = 0.0001) and *INFB* (*p* = 0.01) groups. Similarly, fever duration was significantly shorter in the *INFA* group than in the VPN (*p* = 0.004) and *INFB* (*p* = 0.02) groups. The duration of myalgia was significantly longer in the VPN group compared with the *INFA* group (*p* = 0.04) and those with other etiologies (*p* = 0.01). Additionally, the interval between fever onset and myalgia onset was significantly longer in the *INFB* group than in the *INFA* and VPN groups (Table 5).

To reduce heterogeneity, patients with other etiologies were excluded, and the analysis was repeated including only the VPN, *INFA*, and *INFB* groups. In this restricted analysis, patients with INFA-associated BACM had significantly shorter recovery times (*p* = 0.04), lower median CK levels (*p* = 0.0001), shorter fever duration (*p* = 0.01), and a shorter interval between fever onset and myalgia onset (*p* = 0.02) compared with those in the *INFB* and VPN groups (Table 6).

### 4.5. Factors Associated with Hospitalization

The results of the logistic regression analysis identifying factors associated with hospitalization are presented in Table 7. Elevated CK levels were independently associated with hospitalization, with increasing CK values corresponding to a higher likelihood of admission (OR, 2.72 per 1000 U/L increase; 95% CI, 1.00–7.39; *p* = 0.05). Inability to walk at presentation was also independently associated with hospitalization, increasing the risk of admission by nearly threefold (OR, 2.95; 95% CI, 0.82–10.61; *p* = 0.049). No significant associations were observed between hospitalization and age, sex, or the presence of myalgia (*p* > 0.05) (Table 7).

### 4.6. Treatment and Outcome

Treatment modalities included antiviral therapy (oseltamivir), hydration therapy, urine alkalinization, hemodialysis, and nonsteroidal anti-inflammatory drugs. Intravenous hydration was administered significantly more frequently in hospitalized patients than in outpatients (*p* < 0.05) (Table 3). Urine alkalinization was required in four hospitalized patients. One patient developed acute kidney injury and required admission to the pediatric intensive care unit, where hemodialysis was initiated.

Recovery time was significantly longer in hospitalized patients than in outpatients (*p* < 0.05). Recurrence of BACM occurred in 11 patients (11.8%), with no significant difference between outpatient and hospitalized groups (9.5% vs. 13.7%, *p* = 0.74). Among recurrent cases, nine patients experienced two episodes, whereas two patients experienced three episodes. However, the timing of recurrence differed significantly (*p* = 0.04) (Table 3), with hospitalized patients more frequently experiencing recurrence within the same year, whereas outpatient cases tended to recur after a longer interval. The mean CK level during recurrent episodes was 4212 ± 3801 U/L (median 2999 U/L; range 1303–13,855 U/L). Metabolic screening was performed in 10 patients with recurrent BACM; carnitine palmitoyltransferase I deficiency was identified in one patient, whereas no metabolic disorder was detected in the remaining patients. No significant associations were found between recurrence and age, sex, CK levels, myalgia, or inability to walk (Table 8). All patients recovered completely without long-term sequelae.

Rhabdomyolysis occurred in three hospitalized patients. *INFB* was identified in two cases, while viral testing was negative in one case. One patient required hemodialysis. All patients recovered fully without sequelae.

## 5. Discussion

In this 10-year single-center retrospective study, we evaluated the clinical, laboratory, and etiological characteristics of children diagnosed with benign acute childhood myositis (BACM) and identified factors associated with hospitalization. Overall, our findings confirm that BACM is a seasonal and predominantly self-limiting condition; however, a substantial proportion of patients required hospitalization. Elevated CK levels and inability to walk at presentation were independently associated with hospitalization, whereas demographic characteristics and most accompanying symptoms showed no significant association. Furthermore, etiological differences were associated with variations in disease severity, with *INFA*-associated BACM showing a milder clinical course and VPN cases more frequently requiring hospitalization. Importantly, our findings also highlight the relevance of regional viral epidemiology, as SFV was identified in two siblings hospitalized during the summer months. Both patients presented with severe lower extremity symptoms and markedly elevated CK levels but recovered fully with supportive care and intravenous hydration. These cases, previously reported in the literature [7], underscore the importance of considering sandfly fever in the differential diagnosis of BACM, particularly in endemic regions during the summer.

In line with previous studies, we observed a marked male predominance (76.3%) and a mean age of 7.4 ± 3.3 years, consistent with the reported peak incidence of BACM between 6 and 9 years of age [3,8,9,10]. Although the underlying mechanisms remain unclear, previous experimental studies have suggested that influenza viruses may preferentially affect immature muscle cells, which could partly explain the observed age predilection. The male predominance has been attributed to differences in muscle mass, hormonal factors, and possible genetic susceptibility [9,11,12,13,14,15,16]. In our cohort, nearly all patients presented with lower extremity pain, and three-quarters experienced difficulty walking, which likely explains the high rate of (57.0%) ED admissions and reflects the acute functional impairment associated with the disease.

Seasonal distribution in our study demonstrated a clear winter predominance, with most cases occurring in January and February. This pattern is consistent with studies from temperate regions but contrasts with reports from other geographic areas, where peak incidence has been observed during different months [17,18,19]. Such variability likely reflects regional differences in viral circulation, climate, and study design [2,4,5,8,9,11,12,13,20]. Notably, we observed a marked increase in BACM cases in 2024, similar to reports describing a post-COVID-19 rise in pediatric viral infections and BACM incidence, possibly related to the concept of “immunity debt” following pandemic-related restrictions [4,20]. Taken together, these findings support the strong association between BACM incidence and seasonal viral circulation.

When outpatient and hospitalized patients with BACM were compared, no significant differences were observed in demographic characteristics, including age and sex, or clinical features such as duration of fever, cough, URTI, myalgia, sore throat, and additional complaints (diarrhea, abdominal pain, headache, vomiting, conjunctivitis, postnasal drip and somnolence). Likewise, the interval between fever onset and myalgia onset did not differ significantly between the two groups. However, inability to walk, which reflects functional impairment, was significantly more frequent among hospitalized patients in our study, underscoring its role as a marker of functional severity and clinical concern. This finding aligns with previous studies reporting higher admission rates among patients presenting with marked gait disturbance or complete loss of ambulation [8,21]. The absence of an association between myalgia alone and hospitalization suggests that functional impairment, rather than pain severity per se, may be more influential in admission decisions. In addition to functional impairment, laboratory markers may also provide objective indicators of disease severity in BACM. Marked elevation of CK is the most characteristic laboratory finding in BACM and reflects the degree of skeletal muscle involvement. In a recent scoping review, Majava et al. reported considerable variability in CK levels across studies, with mean values ranging from 1185 to 14,319 U/L [22]. In our study, CK levels were significantly higher in hospitalized patients, and logistic regression analysis confirmed CK elevation as an independent predictor of hospitalization. Although the odds ratio per 1-unit increase appears small, CK values in BACM typically reach several thousand units per liter; therefore, even incremental increases may translate into clinically meaningful differences in disease severity. These findings are consistent with previous studies identifying CK elevation as an objective parameter associated with admission decisions [5,10].

The association between elevated CK levels and hospitalization highlights the value of CK as an objective marker of muscle involvement in BACM. Although the condition is typically self-limiting, markedly elevated CK levels may prompt closer monitoring because of concerns regarding potential complications such as rhabdomyolysis and secondary renal injury. However, CK should not be interpreted in isolation but rather in conjunction with clinical findings, particularly functional impairment such as inability to walk. In this context, CK measurement at presentation may assist clinicians in early risk stratification and in identifying patients who may benefit from closer monitoring or inpatient observation. Nevertheless, we acknowledge that hospital admission decisions in retrospective studies may partially overlap with markers of clinical severity, potentially introducing an element of circular reasoning.

In patients with recurrent BACM, mean CK levels were also elevated (4212 ± 3801 U/L), exceeding those observed in patients managed on an outpatient basis but remaining lower than levels in the hospitalized cohort. This pattern may suggest a relationship between greater muscle involvement and disease recurrence, in line with prior studies reporting significantly higher CK levels in recurrent compared with non-recurrent BACM cases [2].

In contrast, inflammatory markers and renal function parameters did not differ significantly between hospitalized and outpatient patients, supporting the notion that BACM is primarily a muscle-limited condition rather than a systemic inflammatory process.

In addition to CK elevation, other laboratory parameters may also reflect the extent of muscle involvement in BACM. In our study, hospitalized patients exhibited significantly higher AST and ALT levels as well as lower platelet counts. AST elevation in BACM is likely related to skeletal muscle injury rather than primary hepatic dysfunction, as AST is also present in muscle tissue. Consistent with this mechanism, transient transaminase elevations have been reported in both viral infections and BACM and are thought to reflect skeletal muscle involvement rather than primary hepatic injury [1,6,8,9,10]. Although these elevations are generally self-limiting, higher transaminase levels may parallel greater muscle injury. Additionally, the lower platelet counts observed in hospitalized patients may indicate more pronounced virus-related bone marrow suppression and may reflect greater systemic involvement in these patients. Although previous studies have reported leukopenia and neutropenia in patients with BACM [9,23,24], we did not categorize patients based on these definitions. Instead, leukocyte and neutrophil counts were compared between hospitalized and outpatient patients, and no significant differences were observed between the groups.

Among patients with an identified etiological agent, *INFA* was the most frequently detected pathogen in the outpatient group, whereas VPN cases were more common among hospitalized patients. Previous studies have reported that no identifiable pathogen can be detected in approximately 5–66% of BACM cases, highlighting the substantial variability in virological confirmation rates [22]. Similarly, Hung et al. reported that no detectable pathogen was identified in 37.1% of BACM patients, underscoring the ongoing challenges in establishing virological confirmation in this condition [6]. However, etiological testing was not performed uniformly across patient groups in our study. Hospitalized patients, who typically presented with more severe clinical findings, were more likely to undergo comprehensive viral and serological investigations, introducing potential selection bias. Furthermore, the respiratory viral panel used in our center was limited to *INFA*, *INFB*, *RSV,* and *SARS-CoV-2*, while broader multiplex PCR assays targeting additional viral pathogens were not routinely available during the study period. Consequently, the VPN category likely included infections caused by pathogens not covered by the limited diagnostic panel. In addition, as a tertiary referral center, our institution frequently evaluates patients later in the course of illness, when declining viral loads may reduce detection rates and increase the likelihood of false negative results. Therefore, the higher hospitalization rate observed in the VPN group in our cohort should be interpreted cautiously, as it may reflect testing patterns, undetected viral etiologies, or a more prolonged clinical course rather than true etiological differences. Viral co-infections were observed in a small number of patients; however, due to the limited number of cases, a separate analysis examining their association with clinical severity, hospitalization, or outcomes was not feasible.

In our study, patients with *INFA*-associated BACM had significantly lower median CK levels and shorter fever duration than those in the VPN and *INFB*-positive groups, suggesting a milder clinical course. However, previous studies have reported conflicting results regarding the relative severity of influenza subtypes. While Öztürk et al. observed higher peak CK levels in *INFA*-associated BACM, Gündüz et al. reported higher hospitalization rates and more severe presentations in patients with *INFA* during the 2019–2020 influenza season [8,25]. These discrepancies may be related to differences in study design, circulating viral strains, and particularly the timing of CK measurement. In this context, the lower CK levels and shorter fever duration observed at presentation in *INFA* patients in our cohort may explain why these cases were more often managed on an outpatient basis.

We also observed that the interval between fever onset and myalgia onset was longer in the *INFB* group, whereas it was shorter in the *INFA* group compared with other etiological groups. Chen et al. previously demonstrated that a prolonged fever-to-myalgia interval is associated with greater symptom severity, including inability to walk and abnormal gait [6]. In this context, the shorter interval observed in *INFA*-positive patients in our cohort further supports the interpretation of a milder clinical course.

Conversely, patients with VPN BACM in our study had a significantly longer duration of myalgia compared with both *INFBA* -positive and other etiological groups. Similarly, Öztürk et al. reported longer recovery times in influenza-negative BACM cases than in *INFA*- and *INFB*-positive patients [8]. Consistent with these findings, VPN cases constituted the most frequent etiological group among hospitalized patients in our cohort, suggesting a possible association between influenza-negative etiology and a more prolonged disease course.

A noteworthy finding of our study was the identification of *SFV* in two hospitalized patients presenting during the summer months. Although uncommon, *SFV*-associated BACM has previously been reported in endemic regions, including Turkey [7]. Both patients exhibited lower extremity symptoms and elevated CK levels and recovered fully with supportive care. *Sandfly fever virus* testing is not part of the standard diagnostic evaluation of BACM and was performed selectively based on seasonal and epidemiological considerations. Therefore, selection bias cannot be excluded, as testing may have been more likely in hospitalized or atypical cases. Given the small number of patients, no conclusions can be drawn regarding disease severity. Nevertheless, this observation highlights the importance of considering regional viral epidemiology in the differential diagnosis of BACM, particularly outside the typical influenza season.

Hospitalization rates for benign acute childhood myositis vary widely in the literature, ranging from 4% to 100%, reflecting heterogeneity in study design, patient populations, admission criteria, and institutional practices [2,3,8,12,26,27,28]. In our cohort, elevated CK levels and inability to walk were independently associated with hospitalization. Similarly, Pasquinucci et al. identified elevated CK as a key predictor of admission, along with older age and renal involvement, while reporting an inverse association between gastrointestinal symptoms and hospitalization in patients with high CK levels [4]. Previous studies have also consistently reported markedly elevated CK levels as a major risk factor for hospitalization, although associations with sex, age, and clinical features such as inability to walk or myalgia have been inconsistent across studies [5,10,21]. In contrast, age, renal function, gastrointestinal symptoms, sex, and myalgia were not associated with hospitalization in our cohort. These findings suggest that objective indicators of muscle involvement, particularly CK elevation and functional impairment such as inability to walk, may play a more important role in admission decisions than demographic characteristics or accompanying symptoms. Importantly, simple clinical assessment combined with CK measurement at presentation may help clinicians identify children with BACM who are more likely to require hospitalization and closer monitoring. Early recognition of these predictors may facilitate risk stratification and support more appropriate clinical decision-making.

Management of BACM was primarily supportive and guided by clinical severity in our study. Hospitalized patients more frequently received intravenous hydration and had longer recovery times than those managed as outpatients. However, the higher frequency of intravenous hydration likely reflects differences in the clinical care setting rather than a direct indicator of disease severity, as intravenous fluids are more readily administered in inpatient settings, whereas outpatient management typically relies on oral hydration. Attanasi et al. recommend hospitalization when oral rehydration is not feasible or when concerning clinical or laboratory findings are present, supporting inpatient care in selected cases [1]. Consistent with this, hospitalized patients in our study had higher CK levels and limited oral intake, which may have contributed to the decision to administer intravenous hydration. Follow-up data indicated that most patients achieved complete clinical and biochemical recovery within a few days.

The recurrence rate of BACM in our cohort was 11.8%, which is within the range reported in previous studies [2,5,6,12]. Recurrence was not associated with age, sex, CK levels, myalgia, or inability to walk and did not differ significantly between hospitalized and outpatient patients. Most patients experienced two episodes, consistent with previous reports describing recurrent BACM as an uncommon but recognized phenomenon. Interestingly, the timing of recurrence differed between groups, with hospitalized patients more frequently experiencing recurrence within the same year. Metabolic evaluation performed in most recurrent cases identified carnitine palmitoyltransferase I deficiency in one patient, whereas no metabolic disorder was detected in the remaining patients. Although metabolic abnormalities were uncommon, this finding suggests that metabolic evaluation may be considered in selected patients with recurrent or atypical presentations, in line with previous reports [1]. Importantly, despite these recurrent episodes, all patients recovered completely without long-term sequelae.

Rhabdomyolysis is the most serious complication of BACM, although it is relatively rare, with an incidence of approximately 3% reported in the literature [29,30,31]. Secondary renal injury may occur due to myoglobinuria, particularly in patients with markedly elevated CK levels, often exceeding 16,000 U/L [31]. Nevertheless, several studies have reported no cases of rhabdomyolysis, underscoring the generally benign and self-limiting course of BACM in most patients [1,2,10,12]. Turan et al. reported rhabdomyolysis in 6.7% of cases, all associated with influenza infection [9]. Similarly, Angelman et al. observed rhabdomyolysis in 3% of patients, predominantly linked to *INFA* and frequently complicated by renal failure. In our cohort, rhabdomyolysis developed in three patients, requiring close monitoring in the pediatric intensive care unit; *INFB* was identified in two cases, while viral testing was negative in one, supporting previous observations that influenza, particularly *INFA*, may be associated with more severe muscle involvement. Despite this, all patients in our cohort recovered fully and were discharged without sequelae.

## 6. Limitation

This study has several limitations. First, its retrospective, single-center design may limit generalizability and is subject to selection and information bias. Second, virological testing was not performed uniformly in all patients, and the respiratory viral panel used in our center was limited, which may have resulted in the underdetection of certain viral pathogens. Third, creatine kinase levels were measured at initial presentation rather than at peak values, which may affect comparisons with studies reporting maximal CK levels. Finally, hospital admission decisions may have been influenced not only by clinical severity but also by physician judgment, parental anxiety, healthcare access, and institutional practices, potentially introducing bias when evaluating predictors of hospitalization. Socioeconomic factors were not systematically recorded and therefore could not be analyzed as potential confounders.

## 7. Conclusions

Although BACM is generally a self-limiting condition, hospitalization may be required in selected cases. In our study, elevated CK levels and inability to walk were the primary factors associated with hospitalization, while etiological differences were associated with variations in the clinical course. These findings may assist clinicians in identifying patients who require closer monitoring. Importantly, the detection of *SFV*-associated BACM in our cohort highlights the role of regional viral epidemiology and underscores the need to consider *SFV* in the differential diagnosis of BACM, particularly during the summer months.

## Figures and Tables

**Table 1:** Demographic characteristics of patients with benign acute childhood myositis (BACM).

Patients (*n* = 93)	Mean ± SD Median (Range)
Age, years	7.4 ± 3.3
	7.0 (2.0–17.0)
Sex (*n*, %)	
Female	22 (23.7)
Male	71 (76.3)
Follow-up setting (*n*, %)	
Outpatient	42 (45.2)
Inpatient ward	48 (51.6)
PICU	3 (3.2)
Place of admission (*n*, %)	
ED	53 (57.0)
Pediatric clinic	29 (31.2)
Neurology clinic	11 (11.8)
Mode of admission (*n*,%)	
Direct admission	87 (93.5)
Referred admission	6 (6.5)

Abbreviations: PICU, pediatric intensive care unit; ED, emergency department.

**Table 2:** Comparison of demographic and clinical characteristics between outpatient and hospitalized patients with BACM.

Patients (*n* = 93)			
Variable	Outpatients (*n*:42)	Hospitalized Patients (*n*:51)	*p* Value *
Age (years) Median (range)	7.6 ± 3.4	7.3 ± 3.2	0.65
Sex (*n*, %) Female Male	7 (16.7) 35 (83.3)	15 (29.4) 36 (70.6)	0.14
Duration of symptoms, days Fever Cough URTI Myalgia Inability to walk Sore throat Interval between fever onset and myalgia onset	3.4 ± 0.2 1.5 ± 0.4 3.9 ± 0.3 1.9 ± 0.1 1.4 ± 0.1 3.4 ± 0.8 1.6 ± 0.2	3.4 ± 0.1 1.8 ± 0.2 3.2 ± 0.2 1.8 ± 0.1 1.3 ± 0.08 2.1 ± 0.1 1.8 ± 0.1	0.98 0.49 0.07 0.37 0.62 0.09 0.37
Monthly distribution of cases (*n*, %) January February March April May June August September October November December	10 (23.8) 11 (26.2) 9 (21.4) 2 (4.8) 2 (4.8) 1 (2.4) 0 (0) 1 (2.4) 2 (4.8) 0 (0) 4 (9.4)	13 (25.5) 13 (25.5) 5 (9.8) 3 (5.9) 2 (3.9) 2 (3.9) 1 (2.0) 2 (3.9) 0 (0) 3 (5.9) 7 (13.7)	0.37
Symptoms Fever (*n*, %) Yes No Cough (*n*, %) Yes No URTI (*n*, %) Yes No Myalgia (*n*, %) Yes No Inability to Walk (*n*, %) Yes No Sore Throat (*n*, %) Yes No ** Additional Complaint (*n*, %) Yes No	41 (97.6) 1 (2.4) 20 (47.6) 22 (52.4) 39 (92.9) 3 (7.1) 41 (97.6) 1 (2.4) 24 (57.1) 18 (42.9) 7 (16.7) 35 (83.3) 10 (23.8) 32 (76.2)	50 (98.0) 1 (2.0) 21 (41.2) 30 (58.8) 48 (94.1) 3 (5.9) 51 (100.0) 0 (0) 46 (90.2) 5 (9.8) 8 (15.7) 43 (84.3) 19 (37.3) 32 (62.7)	0.89 0.53 0.81 0.45 0.0001 * 0.90 0.16

* *p* < 0.05 was considered statistically significant. ** Additional Complaint: Diarrhea, abdominal pain, headache, vomiting, eye redness, postnasal drip. Abbreviations: URTI, upper respiratory tract infection; BACM, benign acute childhood myositis.

**Table 3:** Comparison of laboratory parameters, etiological agents, treatment approaches, and outcomes between outpatient and hospitalized patients with BACM.

Variable	Outpatient (*n* = 42)	Hospitalized Patient (*n* = 51)	*p* Value
Hematological and biochemical parameters			
Hb (g/dL)	12.5 ± 1.2	12.6 ± 1.4	0.87
Leukocyte (/mm^3^)	5407.7 ± 387.8	5540.8 ± 520.8	0.84
Lymphocyte (/mm^3^)	2106.0 ± 202.9	2240.7 ± 160.5	0.59
Neutrophil (/mm^3^)	2629.7 ± 303.4	2708.0 ± 444.9	0.89
Platelet (/mm^3^)	230,309.5 ± 9771.9	199,806.3 ± 9894.6	0.02 *
Blood sugar (mg/dL)	96.5 ± 10.8	98.8 ± 15.2	0.64
Creatinine (mg/dL)	0.46 ± 0.02	0.51 ± 0.03	0.15
AST (U/L) (*n*:86)	117.6 ± 17.7	249.6 ± 56.1	0.001 *
ALT (U/L)	50.5 ± 7.0	89.2 ± 23.1	0.03 *
CK (U/L)	1358.7 ± 145.5	10,658.6 ± 4871.6	0.0001 *
CKMB (U/L) (*n*:15)	-	716.1 ± 421.1	-
CRP (mg/dL)	3.9 ± 0.7	4.7 ± 2.1	0.06
LDH (U/L) (*n* = 23)	433.4 ± 74.9	662.2 ± 185.5	0.53
Urinalysis (*n*, %) Normal Abnormal	20 (100.0) 0 (0)	44 (95.7) 2 (4.3)	0.48
Etiological agents (*n*,%) VPN INFA INFB SARS-CoV-2 EBV INFA + RSV INFA + HSV SFV	4 (18.2) 10 (45.5) 5 (22.7) 2 (9.1) 0 (0) 0 (0) 1 (4.5) 0 (0)	20 (50.0) 6 (15.0) 6 (15.0) 4 (10.0) 1 (2.5) 1 (2.5) 0 (0) 2 (5.0)	0.03 *
Complications (*n*,%) Rhabdomyolysis Yes No Acute renal failure (*n*,%) Yes No	0 (0%) 42 (100) 0 (0) 42 (100)	3 (5.9) 48 (94.1) 1 (2.0) 50 (98.0)	0.24 0.54
Comorbidity (*n*,%) Pneumonia Sinusitis Febrile convulsions	0 (0) 2 (100.0) 0 (0)	4 (57.1) 2 (28.6) 1 (14.3)	0.38
Treatment			
Oseltamivir (*n*,%) Yes No	18 (42.9) 24 (57.1)	30 (58.8) 21 (41.2)	0.12
Hydration (*n*,%) Yes No	0 (0) 42 (100.0)	49 (96.1) 2 (3.9)	0.0001 *
Alkalization (*n*,%) Yes No	0 (0) 42 (100)	4 (7.8) 47 (92.2)	0.12
Hemodialysis (*n*,%) Yes No	0 (0) 42 (100)	1 (2.0) 50 (98.0)	0.54
Analgesic treatment (*n*,%) Yes No	31 (73.8) 11 (26.2)	40 (78.4) 11 (21.6)	0.60
Follow-up (mean ± SD)			
Follow-up visit (days) Recovery (days) Control CK (U/L) Control leukocyte (×10^3^/µL)	7.9 ± 3.1 3.4 ± 1.2 130.6 ± 7.5 7227.5 ± 351.0	8.0 ± 3.9 4.2 ± 1.9 191.9 ± 19.7 7273.4 ± 290.5	0.81 0.03 * 0.01 * 0.91
Recurrence (*n*, %) Yes No	4 (9.5) 38 (90.5)	7 (13.7) 44 (86.3)	0.74
Recurrence number (*n*:11) (*n*, %) 2 3	2 (50.0) 2 (50.0)	7 (100) 0 (0)	0.11
Recurrence timing (years) (*n*, %) Within the same year 1 year ago 2 years ago	0 (0) 1 (25.0) 3 (75.0)	4 (57.1) 2 (28.6) 1 (14.3)	0.04 *

* Statistically significant. Abbreviations: Hb, hemoglobin; AST, aspartate aminotransferase; ALT, alanine aminotransferase; CK, creatine phosphokinase; CKMB, creatine kinase–myocardial band; LDH, lactate dehydrogenase; BACM, benign acute childhood myositis; INFA, iinfluenza A; INFB, Influenza B; EBV, Epstein–Barr virus; RSV, respiratory syncytial virus; HSV, herpes simplex virus; SFV, sandfly fever virus; VPN, viral panel negative.

**Table 4:** Comparison of clinical features and recovery time of patients with BACM by viral etiology.

Etiological Group	Recovery Time, Days (*n* = 61)	CK Level, U/L (*n* = 62)	Fever Duration, Days (*n* = 60)	Myalgia Duration, Days (*n* = 61)	Inability to Walk, Days (*n* = 46)	Interval Between Fever Onset and Myalgia Onset, Days (*n* = 62)
	Mean ± SD	Mean ± SD	Mean ± SD	Mean ± SD	Mean ± SD	Mean ± SD
VPN	4.0 ± 0.3	3547 (451–247,440)	3.6 ± 0.3	2.0 ± 0.2	1.2 ± 0.09	1.8 ± 0.2
INFA	2.9 ± 0.3	535.5 (235–5294)	2.6 ± 0.2	1.5 ± 0.2	1.3 ± 0.2	1.1 ± 0.2
INFB	4.7 ± 1.0	1512 (377–51,800)	4.2 ± 0.6	2.0 ± 0.4	1.7 ± 0.2	2.3 ± 0.5
Others **	4.4 ± 0.4	2558 (180–7000)	2.8 ± 0.4	1.3 ± 0.1	1.1 ± 0.1	1.6 ± 0.4
*p* value	0.07	0.0001 *	0.01 *	0.04 *	0.18	0.04 *

* Statistically significant. ** Others: *SARS-CoV-2* infection, *EBV, INFA + RSV, INFA + HSV,* and *SFV.* Abbreviations: BACM, benign acute childhood myositis; CK, creatine kinase; *INFA, influenza A; INFB, influenza B; EBV, Epstein–Barr virus; RSV, respiratory syncytial virus; HSV, herpes simplex virus; SFV, sandfly fever virus*; VPN, viral panel negative.

**Table 5:** Pairwise comparisons of clinical parameters among etiological groups in patients with BACM.

Etiological Group					
	1	2	3	4	*p*
	VPN (*n* = 22)	INFA (*n* = 16)	INFB (*n* = 11)	Others* (*n* = 11)	
CK level (U/L) (*n* = 62) Median (range)	3547 (451–247,440)	535.5 (235–5294)	1512 (377–51,800)	2558 (180–7000)	0.0001 *
*p* (1–2) 0.0001 *	*p* (1–3) 0.30	*p* (1–4) 0.18	*p* (2–3) 0.01 *	*p* (2–4) 0.08	*p* (3–4) 0.58
Duration of fever (days) (*n* = 60)	3.6 ± 0.3	2.6 ± 0.2	4.2 ± 0.6	2.8 ± 0.4	0.01 *
*p* (1–2) 0.004 *	*p* (1–3) 0.55	*p* (1–4) 0.06	*p* (2–3) 0.02 *	*p* (2–4) 0.79	*p* (3–4) 0.08
Duration of myalgia (days) (*n* = 61)	2.0 ± 0.2	1.5 ± 0.2	2.0 ± 0.4	1.3 ± 0.1	0.04 *
*p* (1–2) 0.04 *	*p* (1–3) 0.73	*p* (1–4) 0.01 *	*p* (2–3) 0.19	*p* (2–4) 0.45	*p* (3–4) 0.07
Interval between fever onset and myalgia onset (days) (*n*:62)	1.8 ± 0.2	1.1 ± 0.2	2.3 ± 0.5	1.6 ± 0.4	0.04 *
P1–2 0.01 *	*p* (1–3) 0.45	*p* (1–4) 0.37	*p* (2–3) 0.02 *	*p* (2–4) 0.14	*p* (3–4) 0.23

* *p* < 0.05 was considered statistically significant. Pairwise comparisons were performed using post hoc tests following one-way ANOVA. Others* *SARS-CoV-2 infection, EBV, INFA + RSV, INFA + HSV,* and *SFV.* Abbreviations: BACM, benign acute childhood myositis; CK, creatine kinase; *INFA, influenza A; INFB, influenza B; EBV, Epstein–Barr virus; RSV, respiratory syncytial virus; HSV, herpes simplex virus; SFV, sandfly fever virus;* VPN, viral panel negative.

**Table 6:** Comparison of clinical parameters according to etiological groups (INFA, INFB, and VPN) in patients with BACM.

Etiological Group	Recovery Days (*n* = 50)	CK Level (U/L) (*n* = 51) Median (Range)	Fever Duration (Days) (*n* = 49)	Myalgia Duration (Days) (*n* = 50)	Duration of Inability to Walk (Days) (*n* = 38)	Interval Between Fever Onset and Myalgia Onset (Days) (*n* = 51)
VPN	4.0 ± 0.3	3547 (451–247,440)	3.6 ± 0.3	2.0 ± 0.2	1.2 ± 0.09	1.8 ± 0.2
INFA	2.9 ± 0.3	535.5 (235–5294)	2.6 ± 0.2	1.5 ± 0.2	1.3 ± 0.2	1.1 ± 0.2
INFB	4.7 ± 1.0	1512 (377–51,800)	4.2 ± 0.6	2.0 ± 0.4	1.7 ± 0.2	2.3 ± 0.5
*p* value	0.04 *	0.0001 *	0.01 *	0.16	0.19	0.02 *

* *p* < 0.05 was considered statistically significant. Abbreviations: BACM, benign acute childhood myositis; CK, creatine kinase; *INFA*, *influenza A*; *INFB*, *influenza B*; VPN, viral panel negative.

**Table 7:** Logistic regression analysis of factors associated with hospitalization in patients with BACM.

Variable	B	S.E.	Wald	df	*p*	Exp (B)	95% CI for Exp B
Constant	−1.024	0.842	1.477	1	0.22	0.359	-
Age (years)	−0.143	0.092	2.400	1	0.12	0.867	0.723–1.039
Sex (Male)	0.364	0.786	0.215	1	0.64	1.439	0.309–6.711
CK level (per 1000 U/L increase)	1.000	0.275	13.272	1	0.0001 *	2.72	1.00–7.39
Myalgia	−0.110	1.374	0.006	1	0.93	0.896	0.061–13.248
Inability to walk	1.081	0.654	2.736	1	0.049 *	2.948	0.819–10.612

* Statistically significant. After logarithmic conversion and rescaling to represent 1000 U/L increments, the adjusted odds ratio was 2.72 (95% CI: 1.000–7.390), indicating that each 1000 U/L increase in CK approximately triples the probability of hospitalization. Footnote CK values were log-transformed and rescaled to represent 1000 U/L increases in the logistic regression model. Abbreviations: BACM, benign acute childhood myositis; CK, creatine kinase.

**Table 8:** Logistic regression analysis of factors associated with recurrence in patients with BACM.

Variable	B	S.E.	Wald	df	*p*	Exp (B)	95% CI for Exp B
Constant	−2.257	0.974	5.367	1	0.02 *	0.105	-
Age (years)	0.090	0.091	0.966	1	0.33	1.094	(0.915–1.308)
Sex (Male)	0.310	0.769	0.163	1	0.69	1.364	(0.302–6.157)
CK level	0.000	0.000	0.039	1	0.84	1.000	(1.000–1.000)
Myalgia	−0.497	1.149	0.149	1	0.70	0.608	(0.049–7.618)
Inability to walk	−0.858	1.458	0.346	1	0.23	0.424	(0.105–1.707)

* *p* < 0.05 was considered statistically significant. Abbreviations: BACM, benign acute childhood myositis; CK, creatine kinase.

## Data Availability

The original contributions presented in this study are included in the article. Further inquiries can be directed to the corresponding author.

## Abbreviations

BACM, benign acute childhood myositis; AST, aspartate aminotransferase; ALT, alanine aminotransferase; CK, creatine kinase; CK-MB, creatine kinase–myocardial band; LDH, lactate dehydrogenase; INFA, influenza A; INFB, influenza B; EBV, Epstein–Barr virus; RSV, respiratory syncytial virus; HSV, herpes simplex virus; SFV, sandfly fever virus; VPN, viral panel negative.
